# *catena*-Poly[[(1,10-phenanthroline)manganese(II)]-μ_3_-5-methyl­isophthalato]

**DOI:** 10.1107/S1600536808036362

**Published:** 2008-11-13

**Authors:** Zhao-Lian Yu, Jiang-Liang Hu

**Affiliations:** a College of Chemistry and Chemical Engineering, Luoyang Normal University, Luoyang 471022, People’s Republic of China

## Abstract

In the title coupound, [Mn(C_9_H_6_O_4_)(C_12_H_8_N_2_)]_*n*_, the Mn^II^ ion is coordinated by two N atoms [Mn—N = 2.273 (3) and 2.305 (2) Å] from a 1,10-phenanthroline ligand and four O atoms [Mn—O = 2.112 (2)–2.343 (3) Å] from three 5-methyl­isophthalate (mip) ligands in a distorted octa­hedral geometry. Each mip dianion acts as a tetra­dentate ligand connecting three Mn ions. The crystal packing exhibits π–π inter­actions [3.599 (2)–3.755 (2) Å] between the centroids of the six-membered rings of neighbouring 1,10-phenanthroline ligands.

## Related literature

For crystal structure of related polymeric compound, see Nie *et al.* (2001 ▶). For details of the coordination abilities of 1,3-benzene­dicarboxyl­ate derivatives, see: Pan *et al.* (2006 ▶); Yang *et al.* (2002 ▶); Ma *et al.* (2008 ▶).
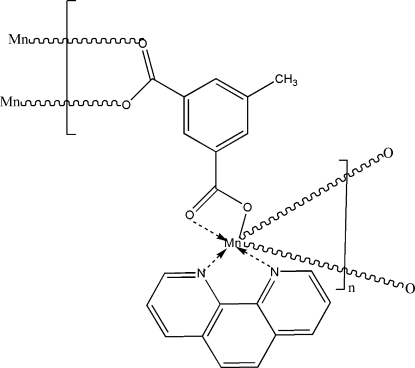


## Experimental

### 

#### Crystal data

[Mn(C_9_H_6_O_4_)(C_12_H_8_N_2_)]
*M* *_r_* = 413.28
Monoclinic, 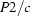

*a* = 9.2837 (11) Å
*b* = 10.3786 (13) Å
*c* = 18.824 (2) Å
β = 101.372 (2)°
*V* = 1778.1 (4) Å^3^
*Z* = 4
Mo *K*α radiation
μ = 0.77 mm^−1^
*T* = 295 (2) K
0.19 × 0.10 × 0.07 mm

#### Data collection

Bruker SMART CCD area-detector diffractometer
Absorption correction: multi-scan (*SADABS*; Bruker, 1997 ▶) *T*_min_ = 0.847, *T*_max_ = 0.948
13275 measured reflections
3313 independent reflections
2230 reflections with *I* > 2σ(*I*)
*R*_int_ = 0.056

#### Refinement

*R*[*F* ^2^ > 2σ(*F* ^2^)] = 0.042
*wR*(*F* ^2^) = 0.097
*S* = 1.02
3313 reflections
254 parameters
H-atom parameters constrained
Δρ_max_ = 0.26 e Å^−3^
Δρ_min_ = −0.24 e Å^−3^

### 

Data collection: *SMART* (Bruker, 1997 ▶); cell refinement: *SAINT* (Bruker, 1997 ▶); data reduction: *SAINT*; program(s) used to solve structure: *SHELXS97* (Sheldrick, 2008 ▶); program(s) used to refine structure: *SHELXL97* (Sheldrick, 2008 ▶); molecular graphics: *SHELXTL* (Sheldrick, 2008 ▶); software used to prepare material for publication: *SHELXTL*.

## Supplementary Material

Crystal structure: contains datablocks I, global. DOI: 10.1107/S1600536808036362/cv2471sup1.cif

Structure factors: contains datablocks I. DOI: 10.1107/S1600536808036362/cv2471Isup2.hkl

Additional supplementary materials:  crystallographic information; 3D view; checkCIF report

## Figures and Tables

**Fig. 1.**
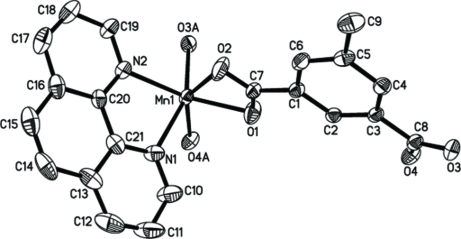
A portion of the title polymeric compound showing the atomic numbering and 40% probability displacement ellipsoids [symmetry code: (A) x,-y+1,z-1/2].

**Fig. 2.**
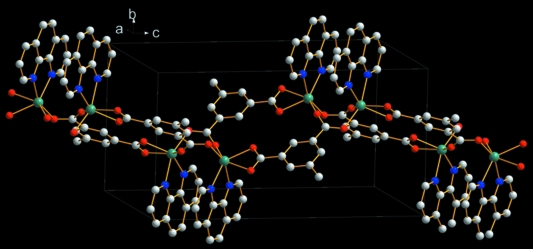
A portion of the stranded polymeric ribbon in (I).

## supplementary crystallographic information

### Comment

The rational design and syntheses of novel coordination polymers have achieved considerable progress in the field of supramolecular chemistry and crystal engineering, owing to their potential applications as gas storage, sensor technology, separation processes, ion exchange, luminescene, magnetism, and catalysis, as well as due to their intriguing variety of architectures and topologies. It has also been proved that careful selection of an appropriate multidentate bridging ligands provides further impetus for the investigation of metal-organic structural architectures as well as potential technological applications. So multicarboxylate acid 1,3-benzenedicarboxylates (H_2_bdc) with variously oriented carboxylate groups have widely been utilized to construct coordination polymers  (Pan *et al.*,  2006; Yang *et al.*,  2002; Ma *et al.*,  2008). Herewith we present the title polymeric compound, (I).

The asymmetric unit of (I) contains one Mn^II^ cation, one phen (= 1,10-phenanthroline) ligand and one mip (H_2_mip=5-methylisophthalic acid) anion. Each Mn ion is octahedrally coordinated by two N atoms of one phen ligand and four oxygen atoms from three mip ligands (Fig. 1). The Mn-N bond lengths are 2.273 (3), 2.305 (2) A° and the Mn-O bond lengths are in the range of 2.112 (2) - 2.343 (3) A°, which are similiar to those reported for nearly isostructural complex (Nie *et al.*,  2001). X-ray structure analysis reveals that (I) consists of 1D chains bridged by mip anions. Each mip anion acts as a tetradentate ligand connecting three Mn ions. Two carboxylic groups of mip ligand have two coordination modes, one bidentately bridging two Mn ions in a syn-syn fashion and the other chelating one Mn ion. Along the (110) direction, the Mn ions are bridged by the mip anions to generate stranded polymeric ribbon containing alternative 8- and 16-membered rings (Fig. 2). Two carboxylic groups bridge two Mn (II) ions to form an 8-membered ring and two mip ligands bridge two Mn (II) ions to form a 16-membered ring. Within the 8-membered ring and 16-membered ring, the Mn—Mn separations are 4.013 (3) and 7.62 (7) A°, respectively.

Close stacking of aromatic rings is observed in the complex, the distances between their centroids from  neighboring phen ligands are 3.599 (2)-3.755 (2) A° (Table 1).

### Experimental

A mixture of 5-methylisophthalic acid (0.2 mmol, 37 mg), phen (0.1 mmol, 21 mg) and Mn(OAc)_2_.4H_2_O (0.1 mmol, 24 mg) in distilled water (15 mL) and ethanol (1mL) was placed in a Teflon-lined stainless steel vessel, heated to 120 ° for 4 days, and then cooled to room temperature over 48 h. Yellow block crystals of (I) were obtained.

### Refinement

The H atoms were positioned geometrically (C—H 0.93-0.96Å), and treated as riding on their parent atoms, with  U_iso_(H) = 1.2-1.5U_eq_(C).

### Crystal data

**Table:**

[Mn(C_9_H_6_O_4_)(C_12_H_8_N_2_)]	*F*_000_ = 844
*M**_r_* = 413.28	*D*_x_ = 1.544 Mg m^−^^3^
Monoclinic, *P*2/*c*	Mo *K*α radiation λ = 0.71073 Å
*a* = 9.2837 (11) Å	Cell parameters from 1788 reflections
*b* = 10.3786 (13) Å	θ = 2.3–19.8º
*c* = 18.824 (2) Å	µ = 0.77 mm^−^^1^
β = 101.372 (2)º	*T* = 295 (2) K
*V* = 1778.1 (4) Å^3^	Block, yellow
*Z* = 4	0.19 × 0.10 × 0.07 mm

### Data collection

**Table:**

Bruker SMART CCD area-detector diffractometer	3313 independent reflections
Radiation source: fine-focus sealed tube	2230 reflections with *I* > 2σ(*I*)
Monochromator: graphite	*R*_int_ = 0.056
*T* = 295(2) K	θ_max_ = 25.5º
φ and ω scans	θ_min_ = 2.3º
Absorption correction: multi-scan(SADABS; Bruker, 1997)	*h* = −11→11
*T*_min_ = 0.847, *T*_max_ = 0.948	*k* = −12→12
13275 measured reflections	*l* = −22→22

### Refinement

**Table:**

Refinement on *F*^2^	Secondary atom site location: difference Fourier map
Least-squares matrix: full	Hydrogen site location: inferred from neighbouring sites
*R*[*F*^2^ > 2σ(*F*^2^)] = 0.042	H-atom parameters constrained
*wR*(*F*^2^) = 0.097	*w* = 1/[σ^2^(*F*_o_^2^) + (0.0334*P*)^2^ + 0.7839*P*] where *P* = (*F*_o_^2^ + 2*F*_c_^2^)/3
*S* = 1.02	(Δ/σ)_max_ < 0.001
3313 reflections	Δρ_max_ = 0.26 e Å^−^^3^
254 parameters	Δρ_min_ = −0.24 e Å^−^^3^
Primary atom site location: structure-invariant direct methods	Extinction correction: none

### Special details

**Table:**

Geometry. All esds (except the esd in the dihedral angle between two l.s. planes) are estimated using the full covariance matrix. The cell esds are taken into account individually in the estimation of esds in distances, angles and torsion angles; correlations between esds in cell parameters are only used when they are defined by crystal symmetry. An approximate (isotropic) treatment of cell esds is used for estimating esds involving l.s. planes.
Refinement. Refinement of F^2^ against ALL reflections. The weighted R-factor wR and goodness of fit S are based on F^2^, conventional R-factors R are based on F, with F set to zero for negative F^2^. The threshold expression of F^2^ > 2sigma(F^2^) is used only for calculating R-factors(gt) etc. and is not relevant to the choice of reflections for refinement. R-factors based on F^2^ are statistically about twice as large as those based on F, and R- factors based on ALL data will be even larger.

### Fractional atomic coordinates and isotropic or equivalent isotropic displacement parameters (Å^2^)

**Table:**

	*x*	*y*	*z*	*U*_iso_*/*U*_eq_	
Mn1	0.36904 (5)	0.31882 (4)	0.32302 (2)	0.03865 (16)	
O1	0.3711 (3)	0.4040 (3)	0.42907 (12)	0.0685 (8)	
O2	0.1789 (3)	0.2896 (3)	0.38683 (13)	0.0757 (8)	
O3	0.2707 (2)	0.5464 (2)	0.74411 (11)	0.0480 (6)	
O4	0.4110 (3)	0.6197 (2)	0.67041 (11)	0.0548 (6)	
N1	0.4764 (3)	0.1298 (3)	0.36551 (15)	0.0520 (7)	
N2	0.2489 (3)	0.1543 (2)	0.25312 (13)	0.0443 (7)	
C1	0.1932 (3)	0.3809 (3)	0.50355 (15)	0.0385 (7)	
C2	0.2745 (3)	0.4529 (3)	0.55968 (15)	0.0362 (7)	
H2	0.3655	0.4860	0.5554	0.043*	
C3	0.2200 (3)	0.4753 (3)	0.62196 (15)	0.0353 (7)	
C4	0.0818 (3)	0.4276 (3)	0.62701 (16)	0.0407 (8)	
H4	0.0450	0.4437	0.6687	0.049*	
C5	−0.0017 (3)	0.3569 (3)	0.57130 (17)	0.0441 (8)	
C6	0.0567 (4)	0.3342 (3)	0.51018 (16)	0.0449 (8)	
H6	0.0026	0.2861	0.4724	0.054*	
C7	0.2510 (4)	0.3568 (3)	0.43524 (16)	0.0458 (8)	
C8	0.3075 (4)	0.5517 (3)	0.68310 (16)	0.0387 (7)	
C9	−0.1512 (4)	0.3073 (4)	0.5774 (2)	0.0629 (10)	
H9A	−0.2236	0.3411	0.5383	0.094*	
H9B	−0.1736	0.3344	0.6228	0.094*	
H9C	−0.1516	0.2149	0.5751	0.094*	
C10	0.5876 (4)	0.1195 (4)	0.4209 (2)	0.0795 (13)	
H10	0.6301	0.1942	0.4430	0.095*	
C11	0.6440 (5)	−0.0001 (5)	0.4477 (3)	0.1003 (17)	
H11	0.7215	−0.0045	0.4873	0.120*	
C12	0.5841 (5)	−0.1089 (5)	0.4151 (3)	0.0919 (15)	
H12	0.6213	−0.1887	0.4323	0.110*	
C13	0.4680 (4)	−0.1032 (4)	0.3564 (2)	0.0641 (11)	
C14	0.3982 (5)	−0.2131 (4)	0.3178 (3)	0.0758 (13)	
H14	0.4325	−0.2952	0.3318	0.091*	
C15	0.2853 (5)	−0.1995 (4)	0.2624 (3)	0.0759 (13)	
H15	0.2421	−0.2729	0.2390	0.091*	
C16	0.2290 (4)	−0.0773 (3)	0.2381 (2)	0.0576 (10)	
C17	0.1094 (5)	−0.0582 (5)	0.1816 (2)	0.0809 (13)	
H17	0.0633	−0.1288	0.1565	0.097*	
C18	0.0596 (5)	0.0622 (5)	0.1628 (2)	0.0765 (12)	
H18	−0.0222	0.0746	0.1260	0.092*	
C19	0.1331 (4)	0.1674 (4)	0.19962 (17)	0.0576 (10)	
H19	0.0995	0.2499	0.1861	0.069*	
C20	0.2954 (4)	0.0339 (3)	0.27317 (17)	0.0428 (8)	
C21	0.4156 (4)	0.0209 (3)	0.33261 (18)	0.0475 (9)	

### Atomic displacement parameters (Å^2^)

**Table:**

	*U*^11^	*U*^22^	*U*^33^	*U*^12^	*U*^13^	*U*^23^
Mn1	0.0482 (3)	0.0372 (3)	0.0286 (2)	−0.0040 (2)	0.0030 (2)	−0.0008 (2)
O1	0.0677 (18)	0.093 (2)	0.0506 (15)	−0.0197 (16)	0.0247 (14)	−0.0229 (14)
O2	0.0743 (18)	0.103 (2)	0.0479 (15)	−0.0192 (16)	0.0075 (14)	−0.0337 (15)
O3	0.0631 (15)	0.0485 (14)	0.0331 (12)	−0.0072 (11)	0.0111 (11)	−0.0046 (10)
O4	0.0553 (15)	0.0618 (15)	0.0442 (13)	−0.0182 (13)	0.0023 (11)	−0.0025 (12)
N1	0.0442 (17)	0.0498 (18)	0.0580 (18)	−0.0018 (14)	0.0003 (15)	0.0111 (15)
N2	0.0490 (17)	0.0452 (18)	0.0376 (15)	−0.0063 (13)	0.0055 (13)	−0.0031 (12)
C1	0.0432 (19)	0.0382 (18)	0.0318 (16)	0.0036 (15)	0.0014 (14)	0.0011 (14)
C2	0.0366 (17)	0.0352 (17)	0.0353 (16)	0.0021 (14)	0.0034 (14)	0.0031 (13)
C3	0.0391 (18)	0.0339 (17)	0.0301 (16)	0.0010 (14)	0.0000 (14)	0.0009 (13)
C4	0.0441 (19)	0.0441 (19)	0.0336 (17)	0.0037 (15)	0.0070 (15)	0.0064 (14)
C5	0.0397 (19)	0.0421 (19)	0.046 (2)	−0.0038 (15)	−0.0016 (16)	0.0100 (15)
C6	0.051 (2)	0.0423 (19)	0.0366 (18)	−0.0012 (16)	−0.0038 (16)	−0.0023 (15)
C7	0.053 (2)	0.049 (2)	0.0314 (17)	0.0088 (17)	−0.0008 (16)	−0.0032 (15)
C8	0.044 (2)	0.0355 (18)	0.0336 (17)	0.0004 (15)	0.0019 (15)	0.0017 (14)
C9	0.045 (2)	0.074 (3)	0.066 (2)	−0.016 (2)	0.0016 (18)	0.012 (2)
C10	0.068 (3)	0.074 (3)	0.082 (3)	−0.001 (2)	−0.018 (2)	0.016 (2)
C11	0.077 (3)	0.094 (4)	0.113 (4)	0.017 (3)	−0.023 (3)	0.038 (3)
C12	0.083 (3)	0.069 (3)	0.121 (4)	0.025 (3)	0.014 (3)	0.040 (3)
C13	0.061 (3)	0.050 (2)	0.086 (3)	0.013 (2)	0.030 (2)	0.017 (2)
C14	0.091 (3)	0.031 (2)	0.119 (4)	0.009 (2)	0.054 (3)	0.007 (2)
C15	0.094 (3)	0.046 (3)	0.099 (3)	−0.011 (2)	0.045 (3)	−0.018 (2)
C16	0.070 (3)	0.042 (2)	0.069 (3)	−0.0120 (19)	0.032 (2)	−0.0112 (18)
C17	0.096 (4)	0.071 (3)	0.071 (3)	−0.025 (3)	0.006 (3)	−0.026 (2)
C18	0.076 (3)	0.086 (3)	0.060 (3)	−0.025 (3)	−0.004 (2)	−0.015 (2)
C19	0.064 (2)	0.061 (2)	0.043 (2)	−0.012 (2)	−0.0016 (19)	−0.0031 (17)
C20	0.047 (2)	0.040 (2)	0.0439 (19)	−0.0033 (16)	0.0165 (16)	−0.0064 (15)
C21	0.048 (2)	0.041 (2)	0.060 (2)	−0.0015 (16)	0.0276 (18)	0.0062 (17)

### Geometric parameters (Å, °)

**Table:**

Mn1—O3^i^	2.112 (2)	C5—C6	1.385 (4)
Mn1—O4^ii^	2.120 (2)	C5—C9	1.505 (4)
Mn1—O1	2.180 (2)	C6—H6	0.9300
Mn1—N1	2.273 (3)	C9—H9A	0.9600
Mn1—N2	2.305 (2)	C9—H9B	0.9600
Mn1—O2	2.343 (3)	C9—H9C	0.9600
Mn1—C7	2.595 (3)	C10—C11	1.402 (5)
O1—C7	1.244 (4)	C10—H10	0.9300
O2—C7	1.235 (4)	C11—C12	1.352 (6)
O3—C8	1.262 (3)	C11—H11	0.9300
O3—Mn1^iii^	2.112 (2)	C12—C13	1.385 (6)
O4—C8	1.252 (3)	C12—H12	0.9300
O4—Mn1^ii^	2.120 (2)	C13—C21	1.418 (5)
N1—C10	1.319 (4)	C13—C14	1.435 (5)
N1—C21	1.357 (4)	C14—C15	1.332 (5)
N2—C19	1.327 (4)	C14—H14	0.9300
N2—C20	1.351 (4)	C15—C16	1.414 (5)
C1—C6	1.385 (4)	C15—H15	0.9300
C1—C2	1.390 (4)	C16—C17	1.392 (5)
C1—C7	1.509 (4)	C16—C20	1.410 (4)
C2—C3	1.385 (4)	C17—C18	1.355 (6)
C2—H2	0.9300	C17—H17	0.9300
C3—C4	1.396 (4)	C18—C19	1.397 (5)
C3—C8	1.498 (4)	C18—H18	0.9300
C4—C5	1.386 (4)	C19—H19	0.9300
C4—H4	0.9300	C20—C21	1.423 (4)
			
Cg1···Cg2^iv^	3.709 (2)	Cg3···Cg3^iv^	3.599 (2)
Cg2···Cg2^v^	3.755 (2)		
			
O3^i^—Mn1—O4^ii^	96.85 (9)	O2—C7—C1	119.3 (3)
O3^i^—Mn1—O1	107.46 (10)	O1—C7—C1	119.4 (3)
O4^ii^—Mn1—O1	89.43 (9)	O2—C7—Mn1	64.41 (18)
O3^i^—Mn1—N1	156.59 (10)	O1—C7—Mn1	56.88 (17)
O4^ii^—Mn1—N1	83.61 (9)	C1—C7—Mn1	175.9 (2)
O1—Mn1—N1	95.94 (11)	O4—C8—O3	123.6 (3)
O3^i^—Mn1—N2	89.75 (9)	O4—C8—C3	118.1 (3)
O4^ii^—Mn1—N2	127.49 (9)	O3—C8—C3	118.3 (3)
O1—Mn1—N2	137.66 (10)	C5—C9—H9A	109.5
N1—Mn1—N2	72.00 (10)	C5—C9—H9B	109.5
O3^i^—Mn1—O2	100.46 (9)	H9A—C9—H9B	109.5
O4^ii^—Mn1—O2	145.47 (8)	C5—C9—H9C	109.5
O1—Mn1—O2	56.93 (9)	H9A—C9—H9C	109.5
N1—Mn1—O2	91.74 (10)	H9B—C9—H9C	109.5
N2—Mn1—O2	82.42 (9)	N1—C10—C11	122.4 (4)
O3^i^—Mn1—C7	106.19 (9)	N1—C10—H10	118.8
O4^ii^—Mn1—C7	117.61 (10)	C11—C10—H10	118.8
O1—Mn1—C7	28.55 (9)	C12—C11—C10	119.1 (4)
N1—Mn1—C7	94.06 (10)	C12—C11—H11	120.5
N2—Mn1—C7	110.05 (10)	C10—C11—H11	120.5
O2—Mn1—C7	28.38 (9)	C11—C12—C13	120.8 (4)
C7—O1—Mn1	94.6 (2)	C11—C12—H12	119.6
C7—O2—Mn1	87.2 (2)	C13—C12—H12	119.6
C8—O3—Mn1^iii^	117.0 (2)	C12—C13—C21	117.1 (4)
C8—O4—Mn1^ii^	157.6 (2)	C12—C13—C14	124.9 (4)
C10—N1—C21	118.9 (3)	C21—C13—C14	118.0 (4)
C10—N1—Mn1	124.7 (3)	C15—C14—C13	121.3 (4)
C21—N1—Mn1	116.4 (2)	C15—C14—H14	119.4
C19—N2—C20	118.2 (3)	C13—C14—H14	119.4
C19—N2—Mn1	125.9 (2)	C14—C15—C16	122.1 (4)
C20—N2—Mn1	115.7 (2)	C14—C15—H15	119.0
C6—C1—C2	119.3 (3)	C16—C15—H15	119.0
C6—C1—C7	120.3 (3)	C17—C16—C20	116.8 (4)
C2—C1—C7	120.4 (3)	C17—C16—C15	124.3 (4)
C3—C2—C1	120.0 (3)	C20—C16—C15	118.9 (4)
C3—C2—H2	120.0	C18—C17—C16	120.7 (4)
C1—C2—H2	120.0	C18—C17—H17	119.6
C2—C3—C4	119.5 (3)	C16—C17—H17	119.6
C2—C3—C8	120.4 (3)	C17—C18—C19	118.8 (4)
C4—C3—C8	120.1 (3)	C17—C18—H18	120.6
C5—C4—C3	121.4 (3)	C19—C18—H18	120.6
C5—C4—H4	119.3	N2—C19—C18	122.7 (4)
C3—C4—H4	119.3	N2—C19—H19	118.6
C6—C5—C4	117.8 (3)	C18—C19—H19	118.6
C6—C5—C9	121.6 (3)	N2—C20—C16	122.7 (3)
C4—C5—C9	120.6 (3)	N2—C20—C21	117.8 (3)
C5—C6—C1	122.0 (3)	C16—C20—C21	119.5 (3)
C5—C6—H6	119.0	N1—C21—C13	121.8 (3)
C1—C6—H6	119.0	N1—C21—C20	118.1 (3)
O2—C7—O1	121.3 (3)	C13—C21—C20	120.1 (3)
			
O3^i^—Mn1—O1—C7	91.9 (2)	O4^ii^—Mn1—C7—O2	−171.05 (19)
O4^ii^—Mn1—O1—C7	−171.1 (2)	O1—Mn1—C7—O2	178.9 (3)
N1—Mn1—O1—C7	−87.6 (2)	N1—Mn1—C7—O2	−86.1 (2)
N2—Mn1—O1—C7	−17.9 (3)	N2—Mn1—C7—O2	−13.8 (2)
O2—Mn1—O1—C7	0.6 (2)	O3^i^—Mn1—C7—O1	−96.9 (2)
O3^i^—Mn1—O2—C7	−104.8 (2)	O4^ii^—Mn1—C7—O1	10.1 (2)
O4^ii^—Mn1—O2—C7	14.1 (3)	N1—Mn1—C7—O1	95.0 (2)
O1—Mn1—O2—C7	−0.6 (2)	N2—Mn1—C7—O1	167.3 (2)
N1—Mn1—O2—C7	95.3 (2)	O2—Mn1—C7—O1	−178.9 (3)
N2—Mn1—O2—C7	166.9 (2)	Mn1^ii^—O4—C8—O3	67.5 (7)
O3^i^—Mn1—N1—C10	139.9 (3)	Mn1^ii^—O4—C8—C3	−114.3 (5)
O4^ii^—Mn1—N1—C10	47.4 (3)	Mn1^iii^—O3—C8—O4	9.6 (4)
O1—Mn1—N1—C10	−41.4 (3)	Mn1^iii^—O3—C8—C3	−168.61 (19)
N2—Mn1—N1—C10	−179.7 (3)	C2—C3—C8—O4	17.0 (4)
O2—Mn1—N1—C10	−98.3 (3)	C4—C3—C8—O4	−162.1 (3)
C7—Mn1—N1—C10	−70.0 (3)	C2—C3—C8—O3	−164.7 (3)
O3^i^—Mn1—N1—C21	−43.1 (4)	C4—C3—C8—O3	16.1 (4)
O4^ii^—Mn1—N1—C21	−135.6 (2)	C21—N1—C10—C11	−0.9 (6)
O1—Mn1—N1—C21	135.7 (2)	Mn1—N1—C10—C11	176.0 (3)
N2—Mn1—N1—C21	−2.7 (2)	N1—C10—C11—C12	1.0 (8)
O2—Mn1—N1—C21	78.8 (2)	C10—C11—C12—C13	−0.4 (8)
C7—Mn1—N1—C21	107.1 (2)	C11—C12—C13—C21	−0.2 (7)
O3^i^—Mn1—N2—C19	−17.7 (3)	C11—C12—C13—C14	179.4 (4)
O4^ii^—Mn1—N2—C19	−116.2 (3)	C12—C13—C14—C15	178.9 (4)
O1—Mn1—N2—C19	98.4 (3)	C21—C13—C14—C15	−1.6 (6)
N1—Mn1—N2—C19	177.2 (3)	C13—C14—C15—C16	0.6 (6)
O2—Mn1—N2—C19	82.8 (3)	C14—C15—C16—C17	−178.6 (4)
C7—Mn1—N2—C19	89.4 (3)	C14—C15—C16—C20	0.6 (6)
O3^i^—Mn1—N2—C20	167.8 (2)	C20—C16—C17—C18	−1.0 (6)
O4^ii^—Mn1—N2—C20	69.3 (2)	C15—C16—C17—C18	178.2 (4)
O1—Mn1—N2—C20	−76.1 (3)	C16—C17—C18—C19	2.0 (7)
N1—Mn1—N2—C20	2.7 (2)	C20—N2—C19—C18	−1.1 (5)
O2—Mn1—N2—C20	−91.7 (2)	Mn1—N2—C19—C18	−175.5 (3)
C7—Mn1—N2—C20	−85.1 (2)	C17—C18—C19—N2	−0.9 (6)
C6—C1—C2—C3	1.2 (4)	C19—N2—C20—C16	2.1 (5)
C7—C1—C2—C3	179.5 (3)	Mn1—N2—C20—C16	177.1 (2)
C1—C2—C3—C4	−1.5 (4)	C19—N2—C20—C21	−177.3 (3)
C1—C2—C3—C8	179.4 (3)	Mn1—N2—C20—C21	−2.4 (4)
C2—C3—C4—C5	0.8 (4)	C17—C16—C20—N2	−1.1 (5)
C8—C3—C4—C5	179.9 (3)	C15—C16—C20—N2	179.6 (3)
C3—C4—C5—C6	0.3 (4)	C17—C16—C20—C21	178.4 (3)
C3—C4—C5—C9	−179.4 (3)	C15—C16—C20—C21	−0.9 (5)
C4—C5—C6—C1	−0.6 (5)	C10—N1—C21—C13	0.3 (5)
C9—C5—C6—C1	179.1 (3)	Mn1—N1—C21—C13	−176.9 (2)
C2—C1—C6—C5	−0.1 (5)	C10—N1—C21—C20	179.7 (3)
C7—C1—C6—C5	−178.4 (3)	Mn1—N1—C21—C20	2.5 (4)
Mn1—O2—C7—O1	1.1 (3)	C12—C13—C21—N1	0.3 (5)
Mn1—O2—C7—C1	−178.2 (3)	C14—C13—C21—N1	−179.3 (3)
Mn1—O1—C7—O2	−1.2 (4)	C12—C13—C21—C20	−179.2 (3)
Mn1—O1—C7—C1	178.1 (2)	C14—C13—C21—C20	1.3 (5)
C6—C1—C7—O2	−3.6 (5)	N2—C20—C21—N1	0.0 (4)
C2—C1—C7—O2	178.0 (3)	C16—C20—C21—N1	−179.5 (3)
C6—C1—C7—O1	177.0 (3)	N2—C20—C21—C13	179.4 (3)
C2—C1—C7—O1	−1.3 (5)	C16—C20—C21—C13	−0.1 (5)
O3^i^—Mn1—C7—O2	82.0 (2)		

Symmetry codes: (i) *x*, −*y*+1, *z*−1/2; (ii) −*x*+1, −*y*+1, −*z*+1; (iii) *x*, −*y*+1, *z*+1/2; (iv) −*x*+1, *y*, −*z*+1/2; (v) −*x*, *y*, −*z*+1/2.
